# Body mass index and blood urea nitrogen to creatinine ratio predicts refeeding hypophosphatemia of anorexia nervosa patients with severe malnutrition

**DOI:** 10.1186/s40337-020-00356-7

**Published:** 2021-01-06

**Authors:** Michitaka Funayama, Yu Mimura, Taketo Takata, Akihiro Koreki, Satoyuki Ogino, Shin Kurose

**Affiliations:** 1 Department of Neuropsychiatry, Ashikaga Red Cross Hospital, 284-1 Yobe, Ashikaga-city, Tochigi, 326-0843 Japan; 2 Department of Neuropsychiatry, Keio University School of Medicine, Shinjuku, Tokyo, Japan; 3 Department of Neuropsychiatry, National Hospital Organization Shimofusa Psychiatric Medical Center, Chiba, Japan; 4 Department of Trauma and Critical Care Medicine, Kyorin University School of Medicine, Mitaka, Tokyo, Japan

**Keywords:** Anorexia nervosa, Hypophosphatemia, Refeeding, Blood urea nitrogen to creatinine ratio, Body mass index (BMI), Phosphorus

## Abstract

**Aim:**

To investigate development of refeeding hypophosphatemia during the refeeding period and the extent of the decrease in the serum phosphorus level among anorexia nervosa patients with severe malnutrition.

**Objective:**

The accurate prediction of the severity of refeeding hypophosphatemia in patients with anorexia nervosa during acute treatment is of great importance. Although some predictors were found in previous reports, these studies used binominal data—the presence or absence of hypophosphatemia—as an outcome indicator but not the extent of serum phosphorus level decrease. It is crucial in clinical settings to predict the extent of the serum phosphorus level decrease as well as development of refeeding hypophosphatemia, in particular, for patients with severe malnutrition, who has a higher risk of death.

**Methods:**

We investigated 63 admissions from 37 patients with anorexia nervosa who had severe malnutrition (admission body mass index 11.5 ± 1.6) and carried out a linear discriminant regression analysis for the development of refeeding hypophosphatemia. The extent of the decrease in the serum phosphorus level were investigated using multiple linear regression analysis. Explanatory variables included data upon admission (age, sex, body mass index, blood urea nitrogen to creatinine ratio, albumin, initial serum phosphorus level, anorexia nervosa type, i.e., restrictive or binge-purge) as well as treatment-related indicators (calorie intake, amount of phosphate administered, and rate of weight gain).

**Results:**

Development of refeeding hypophosphatemia and a change in serum phosphorus levels were predicted by body mass index and elevated blood urea nitrogen to creatinine ratio.

**Conclusions:**

Our study found that refeeding hypophosphatemia among patients with severe malnutrition was predicted by a lower body mass index and elevated blood urea nitrogen to creatinine ratio.

## Plain English summary

Refeeding hypophosphatemia is a life-threatening condition that occurs in patients with severe malnutrition, in particular, anorexia nervosa. It is of great importance to predict the risk of refeeding hypophosphatemia in patients with anorexia nervosa during acute treatment. Although some predictors were found in previous reports, these studies used binominal data—the presence or absence of hypophosphatemia—as an outcome indicator but not the extent of serum phosphorus level decrease. Our study found that development of refeeding hypophosphatemia and a change in serum phosphorus levels were related to elevated blood urea nitrogen to creatinine ratio and body mass index.

## Introduction

Refeeding hypophosphatemia is a life-threatening condition that occurs in patients with severe malnutrition, in particular, anorexia nervosa. It is of great importance to predict the risk of refeeding hypophosphatemia in patients with anorexia nervosa during acute treatment. Recently, researchers have identified some risk factors regarding refeeding hypophosphatemia for patients with anorexia nervosa [1–4]. Golden et al. (2013) [1] found that the incidence of hypophosphatemia is associated with body mass index and rate of weight loss prior to admission, but not with caloric intake after admission. Similarly, Redgrave et al. (2015) [2] showed that body mass index, but not the rate of weight gain after admission, predicts development of refeeding hypophosphatemia. Brown et al. (2015) [3] reported that body mass index and levels of serum potassium, albumin, and hemoglobin are predictors of hypophosphatemia during refeeding of patients with severe malnutrition. Meanwhile, Kameoka et al. (2016) [4] reported that elevated blood urea nitrogen to creatinine ratio (BUN/Cr ratio) at admission predicts the incidence of refeeding hypophosphatemia. These studies suggest that data upon admission, i.e., body mass index, the rate of weight loss, hemoglobin, albumin, potassium, and BUN/Cr ratio, could be used to predict the incidence of hypophosphatemia, rather than indicators related to treatment, i.e., caloric intake or the rate of weight gain. These previous studies focused on the development of refeeding hypophosphatemia, that is, whether patients had hypophosphatemia or not as an outcome indicator, and the threshold of hypophosphatemia varies among these reports, ranging from 2.3 mg/dl to 3.0 mg/dl [1–4].

In the clinical setting, it is also important to predict the extent of reduction in the serum phosphorus level during the refeeding period, in particular, for patients at the highest risk for severe malnutrition, who have a risk of death [5]. In the previous studies, the prescribed calorie was used as an indicator of caloric intake after hospitalization, but not the actual amount of caloric intake. In addition, they did not include phosphorus administration as a variable that might be accountable for development of refeeding hypophosphatemia. To address these issues, we conducted a retrospective study using 63 anorexia nervosa patients with severe malnutrition and investigated predictors for the decrease in the serum phosphorus level during the refeeding period, as well as development of refeeding hypophosphatemia. We hypothesized that some of the above-mentioned variables predict the extent of reduction in the serum phosphorus level as well as development of refeeding hypophosphatemia.

## Materials and methods

### Participants

Ethical aspects of this study were reviewed and approved by the Human Research Ethics Committee at the Ashikaga Red Cross Hospital. This study was performed after obtaining informed consent from all participants upon admission. For a patient below the age of 18 years, informed parental consent was also obtained. Diagnosis was based on criteria in the ICD-10, and each patient was diagnosed by two of the three psychiatrists (MF, AK, and TT), each of whom had > 10 years of experience in psychiatry at the time of the study. Participants were recruited from the neuropsychiatric unit in Ashikaga Red Cross Hospital during the period from April 1999 to March 2018. Among 96 admissions with eating disorders that were managed in our unit (F50.0, anorexia nervosa; F50.2, bulimia nervosa), only those with a body mass index of < 16 (68 admissions) were included to investigate refeeding hypophosphatemia, which are considered severe malnutrition among patients with anorexia nervosa [6]. The five patients who discharged themselves from the hospital against medical advice or declined repeated blood tests were excluded. Thus, 63 admissions met the abovementioned criteria and were included in this study. Consecutive admissions with recurrences of anorexia nervosa were included as separate admissions [2, 4] because weight and nutritional status, and thus risk of refeeding syndrome, changed with each admission [2]. In this study, among a total of 37 patients, all of whom were Japanese, 12 had two or more consecutive admissions, which adds up to a total of 63 admissions. However, considering a potential bias toward a correlation, analyses were repeated using only 37 independent patients.

### Collection of patient information

Electronic medical records of eligible participants were retrospectively reviewed. As outcome indicators, the following two measures were used: the extent of the decrease in serum phosphorus levels from admission to the nadir phosphorus level during the first 2 weeks after admission and the development of refeeding hypophosphatemia (< 2.5 mg/dl serum phosphorus). Explanatory variables included data at admission, i.e., age, sex, body mass index, anorexia nervosa subtype (restrictive or binge-purge), data obtained from laboratory tests (BUN/Cr ratio, serum phosphorus and potassium levels, hemoglobin, and albumin), and indicators involving treatment, i.e., the rate of weight gain during the first 7 days, caloric intake, and amount of intravenous phosphate administered. Classification of anorexia nervosa subtype, restrictive or binge-purge (bulimic-type), was carried out because binge-purge behavior often influences serum electrolytes levels through repeated vomiting, in particular, serum potassium level [7]. Body mass index was calculated as the weight of the individual (in kilograms) divided by the square of the height of the individual (in meters). To calculate the rate of weight gain during the first 7 days, we divided the kilograms gained during the first 7 days by the initial weight. Total caloric intake (kilocalories) refers to the average total caloric intake from day 1 through day 7 [4], including both oral intake and intravenous infusion therapy. If the patient ate only half the provided 1200-kcal meal, the actual amount of total caloric intake was reduced to 600 kcal. To accurately investigate the effect of energy intake on an individual patient depending on his or her weight, an indicator of total caloric intake per body weight was used for this analysis (total caloric intake divided by body weight), which is widely used for diet therapy for diabetes mellitus [8]. Intravenous phosphorus administration, which was not measured in previous studies of predictors for refeeding hypophosphatemia, was included in this study and was defined as the average total intravenous phosphorus administration (millimoles) from day 1 through day 7. In our unit, phosphorus administration is done intravenously, not orally, during the first 7 days. To better calculate the effect of intravenous phosphorus supplementation on refeeding syndrome, the amount of intravenous phosphorus administered was divided by total caloric intake, and this value was used in the statistical analysis. This is because intracellular movement of serum phosphorus is dependent on reintroduction of nutrients, which is mediated by surges in insulin [9].

A laboratory panel, including serum phosphorus, was carried out on admission. From the second examination onwards, each blood test was conducted at 7:30 in the morning before breakfast. To precisely identify the nadir serum phosphorus level, the patients underwent serial laboratory tests; 47 admissions (74.6%) had the test again on the second hospital day, 45 (71.4%) on the third hospital day, 45 (71.4%) on the fourth hospital day, 29 (46.0%) on the fifth hospital day, and 34 (53.6%) on the sixth day, and they continued to have blood tests until the serum phosphorus level went up again. We note that 59 of 63 admissions (93.7%) had the second laboratory test within 48 h of the first.

### Protocol for refeeding and intravenous phosphorus administration

The initial caloric prescription for each patient was decided by individual physicians on admission, based on their assessment of the degree of malnutrition, caloric intake preceding admission, and his or her weight. In addition to oral food, intravenous infusion therapy was often used and, less frequently, nasogastric feeding was also carried out. Normally, the total initial caloric prescription consisted of ~ 600–1400 kcal/day and was usually increased by ~ 200 kcal every day. Intravenous phosphorus administration was not routinely carried out but was prescribed when hypophosphatemia was found in the laboratory test at admission or consecutive tests and when hypophosphatemia was expected to occur [10]. More precisely, when phosphorus levels were below 2.5 mg/dl or within the lower half (2.5 to 3.5 mg/dl) of the standard value of serum phosphorus (2.5 to 4.6 mg/dl) [11], intravenous phosphorus administration was carried out. The amount of phosphorus administration was generally started with 10 or 20 mmol/day, which was adjusted depending on levels of serum phosphorus on serial laboratory tests. This procedure was similarly applied for hypokalemia and hypomagnesemia [10].

### Statistical analysis

The development of refeeding hypophosphatemia and the extent of the decrease in the serum phosphorus level were investigated for these 63 admissions. Explanatory variables for these two object variables included demographics (age, sex, anorexia nervosa subtype), data obtained at admission (body mass index, BUN/Cr ratio, serum phosphorus and potassium levels, hemoglobin, and albumin), indicators involving treatment (the rate of weight gain, caloric intake, and amount of intravenous phosphate administered). The correlation matrix for the measured variables is shown in Table 1. No single variable had a correlation of > 0.6 with other variables, indicating that all variables were relatively independent, such that all variables were included in the following analyses. Regarding the serum potassium levels, we compared them between the restrictive and binge-purge types using Student’s t-test.

**Table 1:** Correlation matrix among characteristics of patients with severe malnutrition with respect to development of refeeding hypophosphatemia

	Age	Sex	Restrictive subtype	Body Mass Index	Hemoglobin	Albumin	Serum potassium level	BUN/Cr ratio	Serum phosphorus level at admission	Total energy/kg	Rate of weight gain	Intravenous phosphorus administration
Age	1											
Sex	−0.32	1.00										
Restrictive subtype	0.26	0.11	1.00									
Body Mass Index	−0.01	0.03	0.46	1.00								
Hemoglobin	−0.23	−0.14	−0.18	0.03	1.00							
Albumin	−0.21	0.04	0.07	0.17	0.43	1.00						
Serum potassium level	0.18	0.16	0.50	0.27	0.03	0.25	1.00					
BUN/Cr ratio	0.29	0.21	0.23	−0.22	−0.04	−0.14	0.23	1.00				
Serum phosphorus level at admission	−0.01	−0.03	− 0.23	−0.14	0.36	0.05	0.07	0.13	1.00			
Total energy/kg	0.02	−0.16	−0.16	−0.35	− 0.11	0.01	0.00	0.04	−0.26	1.00		
Rate of weight gain	−0.25	−0.12	− 0.42	−0.08	0.19	0.07	−0.32	−0.29	− 0.11	0.11	1.00	
Intravenous phosphorus administration	0.16	−0.06	0.17	0.04	0.02	−0.23	−0.10	0.18	0.08	−0.58	0.02	1.00

A linear discriminant regression analysis was performed for the development of refeeding hypophosphatemia (< 2.5 mg/dl serum phosphorus). Ten admissions which already had a serum phosphorus level of < 2.5 mg/dl at admission were also included; thus, all 63 admissions were included in this linear discriminant regression analysis. In addition, a multivariable linear regression analysis was used for the decrease in serum phosphorus levels from admission to nadir hypophosphatemia. Considering potential heterogeneity of patients, i.e., inclusion of 2 male patients, patients with bulimia nervosa, and consecutive admissions, the above-mentioned analyses were also repeated using the three additional cohorts: the female group, the restrictive group, and the independent patient group. For the independent patient group, we used only the data of their first admission. Given low statistical power for the last two groups (*n* = 45, 37, respectively), explanatory variables for these groups were limited to those having *p*-values of < 0.1 in the former analyses using all 63 admissions. Regarding outliers, we defined an outlier as a value (residual error for the multiple linear regression analysis or the discriminant score for the linear discriminant regression analysis) that is three standard deviations from the mean in this article. Excel 2010 (Microsoft, Reymond, USA) with add-on Statcel 3 (OMS Ltd., Tokyo, Japan) was used for all statistical analyses. Two-tailed *p*-values are reported, and p-values of <.05 were considered statistically significant.

## Results

Table 2 demonstrates the demographic factors, laboratory data at admission, and treatment indicators for the study group. Of 63 admissions, only 2 (3.2%) were male participants and the remaining 61 (96.8%) were female participants. Regarding the anorexia nervosa subtype, 45 admissions (71.4%) involved the restrictive type, whereas 18 (28.6%) involved the binge-purge type. The average body mass index was extremely low at 11.5 ± 1.6. Although the average serum potassium level at admission was 3.5 ± 0.9 mmol/l, it differed depending on the subtype (Student’s t-test, *p* < .01). The average serum potassium level of admissions with the binge-purge type was 2.8 ± 0.8 mmol/l, whereas that with the restrictive type was 3.8 ± 0.8 mmol/l. The average total caloric intake during the first 7 days was 1233 ± 590 kcal/day. The average intravenous phosphorus administration during the first 7 days was 9.2 ± 6.2 mmol/day. The initial serum phosphorus level was 3.9 ± 1.4 mg/dl, which decreased by 1.3 mg/dl to 2.6 ± 0.8 mg/dl at nadir hypophosphatemia, which was recorded at 3.9 ± 4.0 days after their admission, that is, on hospital day 4.9 ± 4.0.

**Table 2:** Demographic factors, laboratory data at admission, and treatment indicators of admissions with severe malnutrition (*N* = 63)

Characteristic	Value	Normal range for Japanese women in their 30s ^a^
Age	37.2 ± 10.4 years (range: 14 to 59 years)	
Sex (female participants)	96.8%	
Anorexia nervosa subtype (restrictive subtype)	45 (71.4%)	
Weight at admission (kg)	29.1 ± 4.5	
Body mass index at admission	11.5 ± 1.6	14.1–28.9
Hemoglobin (g/dl)	12.0 ± 2.8 (7.45 ± 0.99 mmol/l)	11.4–14.6 (7.07–9.06 mmol/l)
Albumin (g/dl)	3.7 ± 0.7	4.1–5.0
Serum potassium level (mmol/l)	3.5 ± 0.9	3.6–4.7
Blood urea nitrogen (mg/dl)	27.7 ± 24.5 (9.89 ± 8.75 mmol/l)	7.7–17.7 (2.75–6.32 mmol/l)
Creatinine (mg/dl)	0.86 ± 0.56 (0.08 ± 0.05 mmol/l)	0.49–0.76 (0.04–0.04 mmol/l)
Blood urea nitrogen/creatinine ratio at admission	33.6 ± 19.8	
Serum phosphorus level at admission (mg/dl)	3.9 ± 1.4 (1.23 ± 0.45 mmol/l)	3.0–4.5 (0.97–1.45 mmol/l)
Nadir serum phosphorus level (mg/dl)	2.6 ± 0.8 (0.84 ± 0.26 mmol/l)	3.0–4.5 (0.97–1.45 mmol/l)
Total caloric intake during the first 7 days (kcal/day)	1233 ± 590	
Weight gain during the first 7 days (kg)	1.1 ± 1.8	
Weight gain during hospitalization (kg)	2.9 ± 2.5	
Intravenous phosphorus administration during the first 7 days (mmol/day)	9.2 ± 6.2	
Interval between admission and the hospital day of nadir hypophosphatemia (days)	3.9 ± 4.0	
Duration of hospitalization (days)	43.3 ± 32.4	

^a^https://www.mhlw.go.jp/toukei/kouhyo/data-kou18/data12/junkan-h12-5.pdf and https://www.jslm.org/books/guideline/2018/03.pdf

### Development of refeeding hypophosphatemia

Of 63 patients, 29 experienced hypophosphatemia. For the female group (*n* = 61), the restrictive group (*n* = 45), and the independent patient group (*n* = 37), the numbers of patients experienced hypophosphatemia are 28, 24, 16, respectively. Each analysis that evaluates predictors of hypophosphatemia included all participants in each group. The linear discriminant regression analysis for development of refeeding hypophosphatemia using all 63 participants was related to severity of BUN/Cr ratio (*p* = .004), body mass index (*p* = .007), and restrictive subtype (*p* = .0499) (Table 3). The results for this model produced Mahalanobis distances of 3.44 (error rate, 0.18). When repeated using only 61 female admissions, it again showed similar results, in which only BUN/Cr ratio (*p* = .005) and body mass index (p = .007) significantly contributed to development of refeeding hypophosphatemia (Mahalanobis distances of 3.42, error rate of 0.18). When only 45 admissions of restrictive anorexia type were included, the results were similar with severity of BUN/Cr ratio (*p* = .006), body mass index (*p* = .01), and serum phosphorus level at admission (*p* = .047) influencing development of refeeding hypophosphatemia (Mahalanobis distances, 2.58; error rate, 0.21). When repeated with 37 independent participants, BUN/Cr ratio (*P* = .003) significantly contributed to development of refeeding hypophosphatemia (Mahalanobis distances, 3.20; error rate, 0.19). In this analysis, influence of body mass index did not reach statistical significance with *p*-Value of 0.13. All the discriminant scores of our linear discriminant regression analysis are normally distributed (normality test, *p* > 0.05) and there are no outliers in the discriminant scores.

**Table 3:** Linear discriminant regression analysis for development of refeeding hypophosphatemia among patients with severe malnutrition

Variable	p-Value	F-value	D2(−i)	Discrimination coefficient
**Blood urea nitrogen to creatinine ratio**	0.0044	8.9242	2.5175	0.0743
**Body mass index**	0.0065	8.0656	2.6124	−0.9153
**Restrict subtype**	0.0499	4.0380	3.0976	2.5952
Serum phosphorus level at admission	0.0605	3.6891	3.1430	−0.6530
Serum potassium level	0.0956	2.8856	3.2500	0.9395
Rate of weight gain	0.1260	2.4217	3.3133	0.1108
Albumin	0.1408	2.2396	3.3384	−1.0653
Total energy intake/kg	0.2831	1.1771	3.4886	−0.0302
Sex	0.2931	1.1290	3.4956	−2.8982
Intravenous phosphorus administration/total energy intake	0.8191	0.0528	3.6545	15.4027
Age	0.8491	0.0366	3.6570	−0.0089
Hemoglobin	0.8768	0.0243	3.6588	0.0283

### Decrease in serum phosphorus level

Regarding the decrease in serum phosphorus level, the multiple linear regression model explained as much as 80.9% of the observed variance with a *p*-value of <.01 and an F-value of 22.9. It shows that serum phosphorus level at admission (*p* < .001), body mass index (*p* = .02), and BUN/Cr ratio (*p* = .046) predicted the amount of decrease in the serum phosphorus level (Table 4). When repeated using only 61 female admissions, it again showed similar results, in which only serum phosphorus level at admission (*p* < .001) and body mass index (p = .02) predicted the amount of decrease in the serum phosphorus level (*P* < .001, F = 24.6). When only 45 admissions of restrictive anorexia type were included, serum phosphorus level at admission (p < .001) and elevated BUN/Cr ratio (*p* = .04) influenced development of refeeding hypophosphatemia (P < .001, F = 31.2). Similarly, serum phosphorus level at admission (p < .001) and elevated BUN/Cr ratio (*P* = .03) significantly contributed to the amount of decrease in the serum phosphorus level (P < .001, F = 22.3) when repeated with 37 independent participants. All the residual errors of the multiple linear regression analysis are normally distributed (normality test, *p* > 0.05) and there are no outliers in the residual errors.

**Table 4:** Multiple linear regression analysis for the decrease in serum phosphorus level among patients with severe malnutrition

Variable	p-Value	Standard beta	Regression coefficient	Standard error	Lower 95% confidence limit	Upper 95% confidence limit
**Serum phosphorus level at admission**	1.886 × 10^−15^	0.7877	0.9071	0.0799	0.7466	1.0675
**Body mass index**	0.0195	−0.1808	−0.1828	0.0757	−0.3349	− 0.0306
**Blood urea nitrogen/creatinine ratio**	0.0455	0.1452	0.0120	0.0058	0.0002	0.0237
Albumin	0.0514	−0.1363	−0.3339	0.1673	−0.6699	0.0021
Serum potassium level	0.0535	0.1418	0.2571	0.1300	−0.0040	0.5181
Rate of weight gain	0.0645	0.1288	0.0316	0.0167	−0.0020	0.0652
Total energy intake/kg	0.1068	−0.1426	−0.0108	0.0065	−0.0239	0.0024
Hemoglobin	0.2860	0.0777	0.0460	0.0427	−0.0397	0.1318
Restrictive subtype	0.3049	0.0876	0.3146	0.3035	−0.2950	0.9241
Age	0.3582	0.0648	0.0102	0.0110	−0.0119	0.0323
Sex	0.4567	−0.0520	−0.4808	0.6410	−1.7682	0.8066
Intravenous phosphorus administration/total energy intake	0.7604	−0.0247	−4.8280	15.7467	−36.4561	26.8001

The fact that the decrease in serum phosphorus levels was predicted by the serum phosphorus levels at admission reflects our intravenous phosphorus administration method, in which phosphorus administration was not routinely carried out but was prescribed when hypophosphatemia was found in the laboratory test and when hypophosphatemia was expected to occur. Accordingly, the extent of decrease in serum phosphorus levels tended to be large if a patient had a preserved level of serum phosphorus at admission because patients with higher phosphorus levels at admission did not receive intravenous phosphorus administration until their serum phosphorus levels decreased. However, it is notable that body mass index and BUN/Cr ratio significantly contributed to the decrease in serum phosphorus levels even after the variable of serum phosphorus levels at admission was controlled.

## Discussion

Our study showed that development of refeeding hypophosphatemia and a greater decrease in serum phosphorus levels during refeeding period of patient with severe malnutrition was predicted by lower body mass index and elevated BUN/Cr ratio. Although influence of body mass index on development of refeeding hypophosphatemia did not reach statistical significance in the independent patient group, this is probably because of low statistical power and this variable might reach statistical significance in a large-scale study. These findings suggest that body mass index and BUN/Cr ratio are useful when predicting refeeding hypophosphatemia for patients with severe malnutrition. In a previous report on refeeding hypophosphatemia [4], BUN/Cr ratio was used as an indicator for levels of dehydration. Although elevated BUN/Cr ratio is surely influenced by dehydration, causes of elevated BUN/Cr ratio is not specific for dehydration. It is also influenced by other backgrounds, such as protein-energy malnutrition, catabolic state due to starvation, elevated corticosteroid levels, and renal functions, all of which can occur in this cohort. To clarify severity of dehydration, other indicators such as urine specific gravity or orthostatic hypotension are needed. More severe BUN/Cr ratio [4] and lower body mass index [1–3] have already been found to be associated with a greater likelihood of developing refeeding hypophosphatemia, and our study replicated the importance of using BUN/Cr ratio and body mass index as predictors even for patients with severe malnutrition. In fact, The average body mass index for 63 admissions was 11.5 ± 1.6, suggesting that this study group was consistent with the anorexia nervosa with most severe type of malnutrition as classified among other studies that used multivariable regression analysis, in which the body mass indexes ranged from 12.7 to 16.2 [1–4]. The interval between admission and nadir hypophosphatemia was 3.9 days, which is also similar to previous findings of 4.8 days [4] and within the first week of hospitalization [12] for patients with anorexia nervosa. Similarly, patients who are hospitalized with severe burns also show nadir hypophosphatemia between their third and fifth day of hospitalization [13]. The most important finding of the present study is that we found a predictor for the amount of decrease in serum phosphorus levels during the refeeding period, information that might be used to manage refeeding hypophosphatemia in patients with severe malnutrition using electrolyte repletion. Although a lower body mass index led to a substantial decrease in the serum phosphorus level, energy intake was not correlated with the development of refeeding hypophosphatemia or the decrease in the serum phosphorus level. This finding is consistent with recent reports [1, 14–18] that recommend a higher-calorie diet, starting at 1440–2400 kcal/day. The initial serum phosphorus level did not correlate with development of refeeding hypophosphatemia in the present study and in Brown et al.’s study (2015) [1], both of which are the only studies of patients with anorexia nervosa that used the initial serum phosphorus level as a variable.

Why are body mass index and BUN/Cr ratio predictors for refeeding hypophosphatemia? In addition, why doesn’t the initial serum phosphorus level correlate with development of refeeding hypophosphatemia? A relevant clue may lie in the very mechanism behind electrolyte imbalances under prolonged starvation and the syndrome associated with subsequent refeeding. According to Mehanna et al. 2008 [10], phosphorus is severely depleted during the period of starvation, although serum phosphorus concentrations can remain normal because most of phosphorus is stored in the intracellular compartment [19], which contracts during starvation. In addition, renal excretion of phosphorus is reduced during prolonged fasting. Accordingly, although extracellular serum phosphorus levels are seemingly normal, phosphorus might have already been depleted within the whole body at admission, which might explain the reason why the initial serum phosphorus level did not correlate with development of refeeding hypophosphatemia. Rather, lower body mass index and probable co-existing dehydration might represent the amount of phosphorus depletion during the period of starvation. With the change to anabolism on refeeding, the insulin surge due to glycemia causes substantial intracellular uptake of phosphorus for all intracellular processes and for the structural integrity of cell membranes that are associated with the synthesis of glycogen, fat, and protein [10]. In addition, phosphorus is used for activation of many enzymes and second messengers as well as energy storage in the form of adenosine triphosphate (ATP). Thus, the combined effects of notable phosphorus consumption during the refeeding period together with depletion of total body stores during starvation causes extracellular hypophosphatemia [18]. Given these mechanisms, it might be impractical to estimate amount of phosphorus storage by using the initial serum phosphate levels. In contrast, a lower body mass index and BUN/Cr ratio might reflect the depleted amount of phosphorus during the period of starvation. In fact, severe malnutrition and probable dehydration often co-exist in patients with anorexia nervosa [4, 20].

### Limitations

Our study has several limitations that should be considered. First, absence of a standardized protocol for intravenous phosphorus administration appears to represent a substantial limitation in study design and interpretation of data analyses although amount of intravenous phosphate supplementation was controlled for our statistical analyses. Second, the study population was not large with only 63 admissions. Even with this sample size, however, it was remarkable that the prediction of a decrease in the serum phosphorus level could be explained with an adjusted R-squared of 0.81.Third, this study population might have had a selection bias because we recruited only patients with severe malnutrition. Despite this potential bias, our results are generally compatible with previous research indicating that body mass index and BUN/Cr ratio can predict the severity of refeeding hypophosphatemia among patients with anorexia nervosa [1–4]. Fourth, 4 of 63 admissions (6.3%) did not have the second laboratory tests within 48 h. The average interval between admission and the nadir hypophosphatemia, however, was 93.6 h (3.9 days), which was longer than the 72-h time point at which the four admissions had had the second blood test. Lastly, not all biochemical data were covered, e.g., the serum magnesium level that might have influenced the nadir serum level. This should be addressed in future studies.

## Conclusions

Despite the aforementioned limitations, our study demonstrates that body mass index and raised BUN/Cr ratio might predict development of refeeding hypophosphatemia and a change in serum phosphorus levels in patients with severe malnutrition.

## Data Availability

The datasets generated and/or analyzed during the current study are available from the corresponding author (MF) upon request.

## References

[1] Golden NH, Keane-Miller C, Sainani K, Kapphahn CJ (2013). Higher caloric intake in hospitalized adolescents with anorexia nervosa is associated with reduced length of stay and no increased rate of refeeding syndrome. J Adolesc Health.

[2] Redgrave GW, Coughlin JW, Schreyer CC, Martin LM, Leonpacher AK, Seide M, Verdi AM, Pletch A, Guarda AS (2015). Refeeding and weight restoration outcomes in anorexia nervosa: challenging current guidelines. Int J Eat Disord.

[3] Brown CA, Sabel AL, Gaudiani JL, Mehler PS (2015). Predictors of hypophosphatemia during refeeding of patients with severe anorexia nervosa. Int J Eat Disord.

[4] Kameoka N, Iga J, Tamura M, Tominaga T, Kubo H, Watanabe Y, Sumitani S, Tomotake M, Omori T (2016). Risk factors for refeeding hypophosphatemia in Japanese inpatients with anorexia nervosa. Int J Eat Disord.

[5] Meczekalski B, Podfigurna-Stopa A, Katulski K (2013). Long-term consequences of anorexia nervosa. Maturitas.

[6] Harrington BC, Jimerson M (2015). Initial evaluation, diagnosis, and treatment of anorexia nervosa and bulimia nervosa. Am Fam Physician.

[7] Sato Y, Fukudo S (2015). Gastrointestinal symptoms and disorders in patients with eating disorders. Clin J Gastroenterol.

[8] Morino K, Kondo K, Tanaka S, Nishida Y, Nakae S, Yamada Y, Ugi S, Fuse K, Miyazawa I, Ohi A, Nishida K, Kurihara M, Sasaki M, Ebine N, Sasaki S, Katsukawa F, Maegawa H (2019). Total energy expenditure is comparable between patients with and without diabetes mellitus: Clinical Evaluation of Energy Requirements in Patients with Diabetes Mellitus (CLEVER-DM) Study. BMJ Open Diabetes Res Care.

[9] Garber AK, Sawyer SM, Golden NH, Guarda AS, Katzman DK, Kohn MR, Le Grange D, Madden S, Whitelaw M, Redgrave GW (2016). A systematic review of approaches to refeeding hospitalized patients with anorexia nervosa. Int J Eat Disord.

[10] Mehanna HM, Moledina J, Travis J (2008). Refeeding syndrome: what it is, and how to prevent and treat it. BMJ.

[11] Nakamura Y, Anayama M, Makino Y, Tamura K, Nagawasa M (2016). Use of lanthanum carbonate for patients with chronic kidney disease at prdialysis stage 4 and 5. Therapeutic Res.

[12] Ornstein RM, Golden NH, Jacobson MS, Shenker IR (2003). Hypophosphatemia during nutritional rehabilitation in anorexia nervosa: implications for refeeding and monitoring. J Adolesc Health.

[13] Rimaz S, Moghadam AD, Mobayen M, Mohammadi N, Rimaz S, Aghebati R, Jafary Parvar Z, Rad EH (2019). Changes in serum phosphorus level in patients with severe burns: a prospective study. Burns.

[14] Whitelaw M, Gilbertson H, Lam PY, Sawyer SM (2010). Does aggressive refeeding in hospitalized adolescents with anorexia nervosa result in increased hypophosphatemia?. J Adolesc Health.

[15] Garber AK, Michihata N, Hetnal K, Shafer MA, Moscicke AB (2012). A prospective examination of weight gain in hospitalized adolescents with anorexia nervosa on a recommended refeeding protocol. J Adolesc Health.

[16] O’Connor G, Nicholls D (2013). Refeeding hypophosphatemia in adolescents with anorexia nervosa: a systematic review. Nutr Clin Pract.

[17] Garber AK, Mauldin K, Michihata N, Buckelew SM, Shafer MA, Moscicki AB (2013). Higher calorie diets increase rate of weight gain and shorten hospital stay in hospitalized adolescents with anorexia nervosa. J Adolesc Health.

[18] Katzman DK, Garber AK, Kohn M, Golden NH (2014). Refeeding hypophosphatemia in hospitalized adolescents with anorexia nervosa. J Adolesc Health.

[19] Bansal VK, Walker HK, Hall WD, Hurst JW (1990). Serum inorganic phosphorus. Clinical methods: The History, Physical, and Laboratory Examinations. 3rd edition.

[20] Stheneur C, Bergeron S, Lapeyraque AL (2014). Renal complications in anorexia nervosa. Eat Weight Disord.

